# Association of estimated dietary acid load with albuminuria in Japanese adults: a cross-sectional study

**DOI:** 10.1186/s12882-019-1352-8

**Published:** 2019-05-30

**Authors:** Keiko Kabasawa, Michihiro Hosojima, Ribeka Takachi, Kazutoshi Nakamura, Yumi Ito, Akihiko Saito, Norie Sawada, Shoichiro Tsugane, Junta Tanaka, Ichiei Narita

**Affiliations:** 1 Department of Health Promotion Medicine, Niigata University Graduate School of Medical and Dental Sciences, 1-757 Asahimachi-dori, Chuo-ku, Niigata, 951-8510 Japan; 2 Department of Clinical Nutrition Science, Niigata University Graduate School of Medical and Dental Sciences, Niigata, Japan; 3 Department of Food Science and Nutrition, Nara Women’s University Graduate School of Humanities and Sciences, Nara, Japan; 4 Division of Preventive Medicine, Niigata University Graduate School of Medical and Dental Sciences, Niigata, Japan; 5 Department of Applied Molecular Medicine, Kidney Research Center, Niigata University Graduate School of Medical and Dental Sciences, Niigata, Japan; 6 Epidemiology and Prevention Group, Center for Public Health Sciences, National Cancer Center, Tokyo, Japan; 7 Division of Cliniacal Nephrology and Rheumatology, Niigata University Graduate School of Medical and Dental Sciences, Niigata, Japan

**Keywords:** Acid-base imbalance, Albuminuria, East Asian, Nutrition, Potassium

## Abstract

**Background:**

Acid-base imbalance might promote the progression of chronic kidney disease (CKD), but whether nutrient-derived dietary acid load increases the risk of albuminuria or even high normoalbuminuria is unclear.

**Methods:**

A Japanese cohort comprising 3250 men and 3434 women aged 40–97 years with urine albumin-to-creatinine ratio (ACR) < 33.9 mg/mmol or estimated glomerular filtration rate ≥ 15 ml/min/1.73 m^2^ were assessed. We performed a cross-sectional evaluation of the association between net endogenous acid production (NEAP), estimated as dietary protein to potassium content ratio, and the presence of high normoalbuminuria (ACR: 1.13–3.38 mg/mmol) or microalbuminuria.

**Results:**

Median NEAP was 43.4 (interquartile range (IQR): 34.2–53.4) mEq/day in men and 35.0 (IQR: 27.7–43.6) mEq/day in women. Median ACR was 1.11 (IQR: 0.57–2.49) mg/mmol in men and 1.47 (IQR: 0.82–2.83) mg/mmol in women. In multivariate analysis, the adjusted odds ratio of the highest versus lowest NEAP quartile for microalbuminuria was 1.47 (95% confidence interval (CI): 1.08–1.99) in men and 1.54 (95% CI: 1.11–2.14) in women. For high normoalbuminuria or microalbuminuria, the adjusted odds ratio was 1.28 (95% CI: 1.02–1.59) in men and 1.39 (95% CI: 1.11–1.74) in women. From nutrient composition analysis, subjects with the highest potassium intake, but not protein intake, had lower adjusted odds ratios for the presence of microalbuminuria than those in the lowest quartile for potassium intake.

**Conclusions:**

Higher NEAP was associated with albuminuria and its association might negatively relate to potassium intake in an adult Japanese population.

## Background

An association between acid-base imbalance and kidney disease has been suggested in recent decades [1, 2]. Acid-base balance is mainly controlled by kidney function, but could be also affected by the intake of acid-inducing foods [2, 3]. Several studies have reported an association between acid-base imbalance and chronic kidney disease (CKD) using albuminuria including microalbuminuria as an indicator [4–6]. Albuminuria is a known risk factor for cardiovascular disease and all-cause mortality. Recently, high normoalbuminuria (urine albumin-to-creatinine ratio [ACR] > 1.13 mg/mmol (10 mg/g)) has also been suggested to carry a similar risk in the general population [7–9]. Thus, detection of high normoalbuminuria, as well as microalbuminuria, is important for the prevention of CKD, cardiovascular events, and death. However, no previous studies have demonstrated the association between acid-base balance and high normoalbuminuria.

The estimated dietary acid load is derived from an equation that takes into account organic compounds and is referred to as net endogenous acid production (NEAP). NEAP, estimated by the ratio of dietary protein to potassium content, reflects the balance of acid and base precursors in healthy individuals in a steady state [10] and in individuals with CKD [11]. Lower dietary protein intake was shown to be associated with reduction in NEAP [12], and consequently protein has been considered as the major source of nonvolatile acids due to its metabolism to sulfates and other organic acids [13]. Nevertheless, some studies have reported that protein intake alone was less likely to be associated with CKD in terms of the relationship between NEAP and CKD [6, 14]. Moreover, the association between albuminuria and nutrients is not fully understood.

In this context, intake of other nutrients such as potassium, as well as protein, should be reassessed in relation to NEAP and albuminuria in various settings. Hence, this study sought to determine the association of estimated dietary acid load with microalbuminuria and/or high normoalbuminuria, and to evaluate the association between nutrient components in estimated dietary acid load and albuminuria.

## Methods

### Study population

**Fig. 1:**
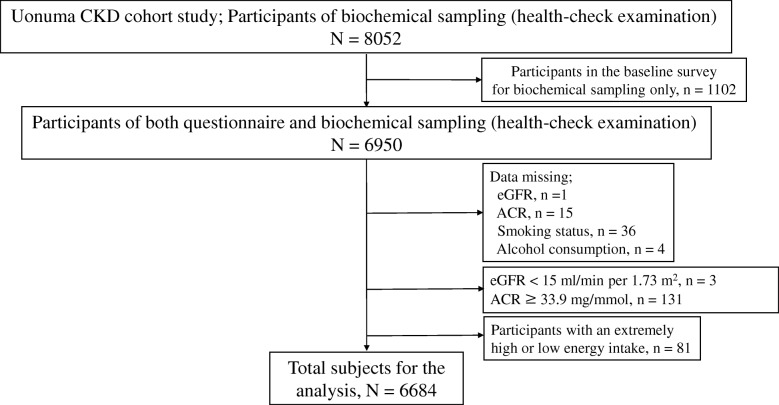
Flowchart of included participants

This cross-sectional study is based on baseline medical examination findings of the Uonuma CKD Cohort Study, which was a population-based prospective cohort study conducted between 2012 and 2015 in the Uonuma region of Niigata Prefecture, Japan, comprising Minamiuonuma City, Uonuma City, and Yuzawa Town [15]. For baseline medical examinations, all 11,406 residents underwent annual local health-check examinations and 8052 of them were subjected to biochemical sampling; 6950 provided urine samples and completed a lifestyle-related questionnaire. We excluded subjects who had low estimated glomerular filtration rate (eGFR) values (< 15 ml/min/1.73 m^2^), macroalbuminuria (ACR ≥ 33.9 mg/mmol), and those who had missing values or incomplete questionnaire data (Fig. 1). Finally, the total number of subjects for analysis in this study was 6684 comprising 3250 men and 3434 women.

### Data collection

During the baseline survey, each participant underwent a health-check examination in the morning or afternoon with or without fasting. The medical examination assessed body weight, body height, blood pressure, fasting or casual plasma glucose, serum creatinine, and glycated hemoglobin (HbA1c), and urine measurements, including urine ACR. Also, self-reported information on antihypertensive or antidiabetic medication was obtained.

Body mass index (BMI) was calculated as body weight (kg) divided by height squared (m^2^). Blood pressure was measured once or twice by using a pressurized cuff on the upper arm at rest in the sitting position. Pulse pressure was calculated as systolic blood pressure minus diastolic blood pressure. Blood glucose was measured by the hexokinase method and HbA1c was measured using high-performance liquid chromatography. Serum creatinine concentration was measured by the enzymatic method. eGFR was obtained by using the following formula modified for Japanese adults [16]: eGFR (ml/min/1.73 m^2^) = 194 × [Serum creatinine (mg/dl)]^-1.094^ × (Age)^-0.287^ × 0.739 (for women). Diabetes was diagnosed based on HbA1c ≥6.5% and fasting plasma glucose ≥7.0 mmol/l or casual plasma glucose ≥11.1 mmol/l, or taking antidiabetic medication. Hypertension was defined as systolic blood pressure ≥ 140 mmHg or diastolic blood pressure ≥ 90 mmHg in accordance with the World Health Organization criteria [17] or alternatively, taking antihypertensive medication.

Urine albumin concentration was measured by the latex agglutination method, and urine creatinine concentration was measured by the enzymatic method in spot urine samples. Albuminuria was evaluated as ACR, which was calculated as urine albumin concentration divided by urinary creatinine concentration. Using ACR cutoff values suggested in a previous study [18], “high normoalbuminuria”, “microalbuminuria”, and “high normoalbuminuria or microalbuminuria” were defined as ACR 1.13–3.38 mg/mmol (10.0–29.9 mg/g), 3.39–33.8 mg/mmol (30.0–299 mg/g), and 1.13–33.8 mg/mmol (10.0–299 mg/g), respectively.

### Assessment of lifestyle and dietary intake

Demographic characteristics, smoking habit, alcohol consumption, physical activity, and food consumption data were obtained from a self-administered questionnaire. A summary of smoking habit, alcohol consumption, and total physical activity can be extracted from the questionnaire [19]. Dietary assessment was based on a validated food frequency questionnaire (FFQ) [20]. For the validation, Spearman’s rank correlation coefficients were calculated for energy-adjusted values between intakes based on the FFQ and 12-day weighed food records. Spearman’s rank correlation coefficients for protein and potassium intake were 0.40 and 0.48 in men and 0.33 and 0.54 in women, respectively.

Estimated dietary acid load was evaluated using the NEAP and the potential renal acid load (PRAL). NEAP and PRAL were derived using a previously published equation: NEAP (mEq/day) = 54.5 × protein (g/day)/potassium (mEq/day) − 10.2 [10]. PRAL (mEq/day) = 0.4888 × protein intake (g/day) + 0.0366 × phosphorus (mg/day) − 0.0205 × potassium (mg/day) − 0.0125 × calcium (mg/day) − 0.0263 × magnesium (mg/day) [21]. Energy-adjusted intakes of protein, potassium, and other specified nutrients or food groups were determined by the residual method [22] after excluding subjects with extreme energy intake (> or < 3 standard deviations [SD] from the mean). NEAP and PRAL estimated from these equations have been validated against based on 24-h urine samples in adolescents and adults [12, 23]. The detailed procedure of the questionnaire survey has been described elsewhere [15, 24].

### Statistical analysis

Characteristics of the subjects are presented as means ± SD, medians (interquartile range [IQR]) or numbers (percentages). Differences in characteristics between men and women were analyzed using the Wilcoxon rank-sum test for continuous variables and the chi-squared test for categorical variables. The unadjusted trend association between NEAP quartile and covariates including potential confounding variables was tested for by using the linear regression model for continuous covariates or the logistic regression model for categorical covariates (yes/no) assigning the NEAP quartile as a continuous variable (Table 1).

**Table 1:** Basal and dietary characteristics according to quartiles of net endogenous acid production, by sex

	Quartile of net endogenous acid production^a^, Men				*P value for linear trend*	Quartile of net endogenous acid production, Women				*P value for linear trend*
	Q1 (< 34.2 mEq/day)	Q2 (34.2 to 43.3 mEq/day)	Q3 (43.4 to 53.3 mEq/day)	Q4 (≥53.4 mEq/day)		Q1 (< 27.7 mEq/day)	Q2 (27.7 to 34.9 mEq/day)	Q3 (35.0 to 43.5 mEq/day)	Q4 (≥43.6 mEq/day)	
N	813	812	812	813		858	859	859	858	
PRAL^b^, mEq/day	−18.7 (−27.5, −12.6)	−2.2 (−5.6, 0.8)	7.0 (4.6, 9.6)	17.7 (14.0, 23.5)	< 0.0001	−32.8 (−44.1, − 25.3)	−14.6 (− 18.6, −11.0)	−2.9 (−6.0, 0.1)	10.1 (6.1, 16.7)	< 0.0001
Age, years	70.1 ± 9.8	69.8 ± 10.0	68.6 ± 10.3	68.2 ± 10.8	< 0.0001	67.7 ± 8.8	68.6 ± 8.9	67.9 ± 9.8	68.8 ± 10.8	0.0613
Body mass index, kg/m^2^	22.9 ± 2.7	22.9 ± 2.9	22.8 ± 2.8	22.8 ± 2.9	0.2473	22.2 ± 3.1	22.6 ± 3.1	22.4 ± 3.3	22.5 ± 3.4	0.1091
Total physical Activity^c^, MET-hour/day	42.0 (37.2, 49.0)	41.3 (36.8, 47.8)	40.8 (36.8, 48.3)	41.3 (36.1, 48.3)	0.0894^d^	39.9 (36.9, 45.2)	39.0 (36.1, 43.8)	38.6 (36.0, 43.2)	38.3 (35.4, 43.5)	< 0.0001^d^
Smoking status					< 0.0001					0.4917
Never-smoker	194 (23.9)	175 (21.6)	178 (21.9)	152 (18.7)		750 (87.4)	768 (89.4)	755 (87.9)	743 (86.6)	
Past smoker	458 (56.3)	476 (58.6)	425 (52.3)	428 (52.6)		60 (7.0)	69 (8.0)	67 (7.8)	69 (8.0)	
Current smoker	161 (19.8)	161 (19.8)	209 (25.7)	233 (28.7)		48 (5.6)	22 (2.6)	37 (4.3)	46 (5.4)	
Alcohol consumption, g ethanol/week					< 0.0001					0.5289
None or rarely	221 (27.2)	178 (21.9)	180 (22.2)	194 (23.9)		566 (66.0)	551 (64.1)	573 (66.7)	595 (69.4)	
1–149	223 (27.4)	226 (27.8)	196 (24.1)	180 (22.1)		240 (28.0)	252 (29.3)	223 (26.0)	208 (24.2)	
150–299	168 (20.7)	192 (23.7)	191 (23.5)	170 (20.9)		34 (4.0)	36 (4.2)	40 (4.7)	31 (3.6)	
300–449	115 (14.2)	129 (15.9)	122 (15.0)	128 (15.7)		13 (1.5)	12 (1.4)	15 (1.8)	15 (1.8)	
450-	86 (10.6)	87 (10.7)	123 (15.2)	141 (17.3)		5 (0.6)	8 (0.9)	8 (0.9)	9 (1.1)	
eGFR, ml/min/1.73 m^2^	73.8 ± 15.3	74.3 ± 15.4	74.5 ± 16.1	75.1 ± 16.2	0.1098	74.3 ± 15.1	74.6 ± 14.4	74.6 ± 15.2	75.2 ± 16.2	0.2309
Urine albumin creatinine ratio, mg/mmol	1.02 (0.57, 2.37)	1.13 (0.57, 2.43)	1.02 (0.57, 2.37)	1.13 (0.68, 2.94)	0.0301^d^	1.36 (0.79, 2.49)	1.47 (0.79, 2.71)	1.47 (0.83, 3.05)	1.58 (0.90, 3.16)	< 0.0001^d^
Systolic blood pressure, mmHg	130.7 ± 18.2	131.5 ± 18.0	130.0 ± 17.7	131.6 ± 18.1	0.7031	127.6 ± 17.8	129.1 ± 18.2	128.4 ± 17.3	128.9 ± 18.3	0.2208
Diastolic blood pressure, mmHg	76.2 ± 11.2	76.0 ± 10.8	76.6 ± 10.8	77.4 ± 10.7	0.0147	73.1 ± 10.8	73.4 ± 10.6	73.6 ± 10.3	72.9 ± 10.7	0.8475
Pulse pressure, mmHg	54.5 ± 13.6	55.5 ± 13.4	53.4 ± 13.7	54.1 ± 13.8	0.1494	54.5 ± 12.9	55.7 ± 13.6	54.9 ± 13.1	56.0 ± 13.7	0.0723
Hypertension	444 (54.6)	479 (59.0)	456 (56.2)	473 (58.2)	0.3108	371 (43.2)	391 (45.5)	394 (45.9)	415 (48.4)	0.0387
Diabetes mellitus	79 (9.7)	69 (8.5)	69 (8.5)	74 (9.1)	0.6803	38 (4.4)	34 (4.0)	39 (4.5)	36 (4.2)	0.9699
Nutritients^e^										
Energy, kcal/day	2001.3 (1605.5, 2505.7)	2069.6 (1661.7, 2564.4)	2089.1 (1703.3, 2591.0)	2032.7 (1569.6, 2680.4)	0.0809^d^	1750.7 (1378.1, 2230.2)	1818.2 (1457.0, 2252.3)	1826.0 (1466.1, 2307.9)	1772.7 (1389.0, 2422.2)	0.0490^d^
Protein, g/day	68.4 ± 14.1	70.9 ± 14.9	70.9 ± 14.4	71.6 ± 19.5	0.0001	67.5 ± 11.5	72.3 ± 11.5	74.9 ± 11.7	77.5 ± 16.3	< 0.0001
Animal protein, g/day	26.4 (18.8, 35.2)	32.0 (22.8, 41.3)	33.8 (25.1, 44)	37.0 (24.4, 50.3)	< 0.0001^d^	27.2 (20.0, 34.8)	34.1 (27.3, 41.6)	38.9 (31.3, 47.3)	43.5 (33.1, 55.5)	< 0.0001^d^
Plant protein, g/day	40.3 ± 7.9	37.1 ± 8.1	35.3 ± 7.9	32.1 ± 8.7	< 0.0001	38.8 ± 7.1	36.5 ± 7.4	35.1 ± 8.7	32.2 ± 9.1	< 0.0001
Fat, g/day	53.3 ± 19.2	54.3 ± 18.5	54.0 ± 17.5	54.3 ± 22.8	0.3476	57.7 ± 15.8	60.0 ± 14.5	62.5 ± 15.2	64.7 ± 19.3	< 0.0001
Carbohydrate, g/day	309.5 ± 50.2	295.2 ± 48.4	289.3 ± 50.0	276.7 ± 62.4	< 0.0001	287.6 ± 37.8	272.3 ± 35.1	259.9 ± 37.2	246.6 ± 48.9	< 0.0001
Potassium, mg/day	3904.8 ± 956.1	3074.0 ± 666.9	2599.2 ± 542.4	2039.1 ± 616.5	< 0.0001	4539.0 ± 949.9	3697.8 ± 598.7	3238.4 ± 522.7	2591.3 ± 636.8	< 0.0001
Calcium, mg/day	617.7 (482.4, 792.5)	538.3 (415.8, 694.1)	469.5 (362.0, 619.4)	355.0 (251.2, 484.5)	< 0.0001^d^	706.8 (576.2, 885.2)	665.1 (539.9, 829.9)	626.0 (488.2, 788)	493.3 (374.6, 672.4)	< 0.0001^d^
Magnesium, mg/day	404.4 ± 99.3	338.0 ± 68.8	296.8 ± 59.2	248.4 ± 59.5	< 0.0001	426.1 ± 94.1	367.1 ± 63.6	333.9 ± 59.2	278.5 ± 58.0	< 0.0001
Phosphorus, mg/day	1192.4 ± 274.8	1169.6 ± 295.0	1107.3 ± 251.1	1042.7 ± 283.9	< 0.0001	1211.0 ± 242.4	1217.7 ± 241.7	1210.4 ± 246.1	1150.1 ± 265.8	< 0.0001
Salt, g/day	13.4 (10.3, 17.3)	11.5 (9.3, 14.1)	10.9 (8.7, 13.2)	9.8 (7.6, 12.2)	< 0.0001^d^	14 (11.2, 17.5)	12.3 (9.9, 14.9)	11.0 (9.0, 13.5)	10.7 (8.7, 12.9)	< 0.0001^d^
Food groups^e^										
Fish, g/day	44.1 (24.2, 69.9)	55.1 (31.6, 83.7)	58.3 (33.4, 92.2)	56.5 (28.0, 101.4)	< 0.0001^d^	47.1 (28.2, 72.3)	63.9 (38.8, 89.7)	69.6 (42.3, 103.8)	73.6 (39.8, 116.6)	< 0.0001^d^
Meat, g/day	35.8 (18.6, 58.2)	46.8 (26.3, 73.6)	56.0 (30.0, 89.1)	67.0 (35.2, 115.4)	< 0.0001^d^	35.4 (20.5, 54.8)	46.8 (26.8, 71.9)	58.3 (35.6, 84.7)	77.6 (42.6, 121.3)	< 0.0001^d^
Grain, g/day	577.6 ± 170.4	610.7 ± 163.0	640.0 ± 164.9	662.5 ± 200.2	< 0.0001	445.2 ± 134.5	483.2 ± 130.9	491.2 ± 132.8	521.4 ± 155.5	< 0.0001
Legumes, g/day	61.2 (34.5, 103.6)	55.4 (32.7, 94.2)	51.1 (28.2, 81.6)	32.7 (15.2, 62.1)	< 0.0001^d^	61.3 (35.3, 105.8)	66.5 (42.5, 105.8)	67.3 (39.5, 107.1)	52.0 (27.0, 89.3)	< 0.0001^d^
Egg, g/day	18.5 (7.9, 33.8)	21.4 (10.1, 38.0)	22.3 (10.2, 37.4)	23.4 (8.9, 47.1)	< 0.0001^d^	17.1 (7.8, 30.8)	22.2 (12.2, 37.0)	24.2 (12.9, 39.2)	41.0 ± 61.0	< 0.0001^d^
Vegetables, g/day	410.9 (259.6, 601.6)	247.8 (170.7, 315.2)	184.1 (121.9, 249.7)	108.8 (60.4, 164.1)	< 0.0001^d^	558.1 (408.9, 759.6)	365.7 (274.9, 486.5)	277.1 (196.2, 361.9)	177.7 (115.5, 252.8)	< 0.0001^d^
Fruits, g/day	197.4 (101.6, 323.1)	129.9 (67.2, 206.1)	95.8 (48.8, 157.6)	43.6 (13.1, 89.5)	< 0.0001^d^	314.1 (190.9, 469.7)	227.8 (145.7, 326.1)	166.8 (102.0, 247.9)	102.5 (47.0, 165.4)	< 0.0001^d^

*eGFR* estimated glomerular filtration rate, *MET-hour* metabolic equivalent task hours, *PRAL* potential renal acid load

Values are as mean ± SD, median (IQR), or number (%)

^a^Net endogenous acid production (mEq/day) = 54.5 × (protein [g/day]/potassium [mEq/day])-10.2

^b^Potential renal acid load (mEq/day) = 0.4888 × protein (g/day) + 0.0366 × phosphorus (mg/day)-0.0205 × potassium (mg/day)-0.0125 × calcium (mg/day)-0.0263 × magnesium (mg/day)

^c^The number of subjects to be analyzed for physical activity decreased by 5 subjects in men and 8 subjects in women due to missing values

^d^Linear trends for total physical activity, urine albumin creatinine ratio, energy, animal protein, calcium, salt, fish, meat, legume, egg, vegetable, and fruit intake were calculated after conversion to logarithmic form

^e^Nutrients and food groups were adjusted for energy intake by the residual method

Multivariate logistic regression analysis was performed to calculate adjusted odds ratios and 95% confidence intervals (95% CI) for each grade of albuminuria. In these calculations, “Controls” are normoalbuminuria cases (ACR < 1.13 mg/mmol) (Tables 2 and 3). Adjustments were done with potential confounders in three models as follows: Model 1 is adjusted for only age (years, continuous); Model 2 is adjust as for Model 1 plus BMI (kg/m^2^, continuous), physical activity (metabolic equivalent task hour, continuous), smoking status (never-smoker, former smoker, or current smoker), alcohol consumption (< 150 g, 150–299 g, 300–449 g, > 450 g ethanol/week), and energy intake (kcal/day, quartile); and Model 3 is adjusted as for Model 2 plus eGFR (ml/min/1.73 m^2^, continuous), pulse pressure (mmHg, continuous), dietary salt intake (g/day, continuous), diabetes (yes or no), and hypertension (yes or no). For sensitivity analysis, we performed a similar multivariate logistic regression analysis to calculate odds ratios of each grade of albuminuria according to PRAL quartile. Furthermore, to examine the role of the nutrient components of NEAP for albuminuria, the association between dietary protein (including animal and plant protein, respectively) or potassium intake and albuminuria was assessed by a similar multivariate logistic regression analysis, as described above. All analyses were performed with SAS version 9.4 (SAS Institute Inc., Cary, NC).

**Table 2:** Odds ratios (95% CIs) for net endogenous acid production and risk of microalbuminuria (a), high normoalbuminuria (b), and high normoalbuminuria or microalbuminuria (c)

(a)	Quartile of net endogenous acid production				*P for trend*
Men	Q1 (< 34.0 mEq/day)	Q2 (34.0 to 43.4 mEq/day)	Q3 (43.5 to 53.3 mEq/day)	Q4 (≥53.4 mEq/day)	
Cases, n	147	154	151	178	
Control, n	417	411	415	386	
Unadjusted	1.00 (reference)	1.06 (0.82 to 1.38)	1.03 (0.79 to 1.35)	1.31 (1.01 to 1.69)	0.0598
Model 1	1.00 (reference)	1.11 (0.84 to 1.46)	1.14 (0.87 to 1.50)	1.49 (1.14 to 1.95)	0.0047
Model 2	1.00 (reference)	1.11 (0.84 to 1.47)	1.15 (0.87 to 1.53)	1.47 (1.12 to 1.94)	0.0074
Model 3	1.00 (reference)	1.12 (0.83 to 1.50)	1.19 (0.88 to 1.61)	1.47 (1.08 to 1.99)	0.0130
Women	Q1 (< 27.7 mEq/day)	Q2 (27.7 to 34.8 mEq/day)	Q3 (34.9 to 43.1 mEq/day)	Q4 (≥43.2 mEq/day)	
Cases, n	142	171	187	205	
Control, n	354	324	309	291	
Unadjusted	1.00 (reference)	1.32 (1.01 to 1.72)	1.51 (1.16 to 1.97)	1.76 (1.35 to 2.29)	< 0.0001
Model 1	1.00 (reference)	1.20 (0.90 to 1.60)	1.56 (1.18 to 2.08)	1.60 (1.20 to 2.13)	0.0003
Model 2	1.00 (reference)	1.14 (0.85 to 1.52)	1.54 (1.15 to 2.06)	1.57 (1.18 to 2.11)	0.0004
Model 3	1.00 (reference)	1.09 (0.80 to 1.50)	1.65 (1.19 to 2.27)	1.54 (1.11 to 2.14)	0.0014
(b)	Quartile of net endogenous acid production				*P for trend*
Men	Q1 (< 34.0 mEq/day)	Q2 (34.0 to 43.0 mEq/day)	Q3 (43.1 to 53.0 mEq/day)	Q4 (≥53.1 mEq/day)	
Cases, n	238	258	239	256	
Control, n	417	397	416	399	
Unadjusted	1.00 (reference)	1.14 (0.91 to 1.42)	1.01 (0.80 to 1.26)	1.12 (0.90 to 1.41)	0.5284
Model 1	1.00 (reference)	1.15 (0.92 to 1.45)	1.07 (0.85 to 1.35)	1.22 (0.97 to 1.53)	0.1642
Model 2	1.00 (reference)	1.15 (0.91 to 1.44)	1.06 (0.84 to 1.34)	1.19 (0.95 to 1.50)	0.2190
Model 3	1.00 (reference)	1.13 (0.89 to 1.43)	1.06 (0.83 to 1.35)	1.18 (0.93 to 1.51)	0.2391
Women	Q1 (< 27.2 mEq/day)	Q2 (27.2 to 34.6 mEq/day)	Q3 (34.7 to 43.2 mEq/day)	Q4 (≥43.2 mEq/day)	
Cases, n	344	355	359	393	
Control, n	338	327	324	289	
Unadjusted	1.00 (reference)	1.07 (0.86 to 1.32)	1.09 (0.88 to 1.35)	1.34 (1.08 to 1.65)	0.0099
Model 1	1.00 (reference)	1.05 (0.85 to 1.31)	1.11 (0.89 to 1.38)	1.32 (1.06 to 1.65)	0.0124
Model 2	1.00 (reference)	1.04 (0.83 to 1.29)	1.10 (0.88 to 1.37)	1.32 (1.06 to 1.65)	0.0130
Model 3	1.00 (reference)	1.06 (0.84 to 1.33)	1.13 (0.89 to 1.43)	1.34 (1.05 to 1.70)	0.0163
(c)	Quartile of net endogenous acid production				*P for trend*
Men	Q1 (< 34.2 mEq/day)	Q2 (34.2 to 43.3 mEq/day)	Q3 (43.4 to 53.3 mEq/day)	Q4 (≥53.4 mEq/day)	
Cases, n	393	411	391	426	
Control, n	420	401	421	387	
Unadjusted	1.00 (reference)	1.10 (0.90 to 1.33)	0.99 (0.82 to 1.21)	1.18 (0.97 to 1.43)	0.2155
Model 1	1.00 (reference)	1.11 (0.91 to 1.36)	1.07 (0.87 to 1.30)	1.30 (1.07 to 1.59)	0.0212
Model 2	1.00 (reference)	1.11 (0.91 to 1.36)	1.06 (0.87 to 1.30)	1.27 (1.04 to 1.56)	0.0414
Model 3	1.00 (reference)	1.09 (0.88 to 1.35)	1.09 (0.88 to 1.35)	1.28 (1.02 to 1.59)	0.0407
Women	Q1 (< 27.7 mEq/day)	Q2 (27.7 to 34.9 mEq/day)	Q3 (35.0 to 43.6 mEq/day)	Q4 (≥43.7 mEq/day)	
Cases, n	504	533	541	578	
Control, n	354	326	318	280	
Unadjusted	1.00 (reference)	1.15 (0.95 to 1.39)	1.20 (0.98 to 1.45)	1.45 (1.19 to 1.77)	0.0003
Model 1	1.00 (reference)	1.10 (0.90 to 1.34)	1.20 (0.98 to 1.47)	1.41 (1.15 to 1.74)	0.0007
Model 2	1.00 (reference)	1.07 (0.87 to 1.31)	1.19 (0.97 to 1.45)	1.40 (1.14 to 1.72)	0.0010
Model 3	1.00 (reference)	1.08 (0.87 to 1.33)	1.21 (0.97 to 1.50)	1.39 (1.11 to 1.74)	0.0028

Net endogenous acid production = 54.5 × protein (g/day) / potassium (mEq/day) − 10.2

‘Controls’ means normoalbuminuric cases (ACR < 1.13 mg/mmol)

Model 1 was adjusted for age (years, continuous); Model 2 was adjusted as for model 1 plus body mass index (kg/m^2^, continuous), physical activity (metabolic equivalent task hour, continuous), smoking status (never-smoker, former smoker, or current smoker), alcohol consumption (< 150 g, 150–299 g, 300–449 g, > 450 g ethanol/week), energy intake(kcal/day, quartiles); and Model 3 was adjusted as for model 2 plus eGFR (ml/min/1.73 m^2^, continuous), dietary salt intake (g/day, continuous), pulse pressure (mmHg, continuous), diabetes (yes or no), and hypertention (yes or no)

In model 2 and model 3, the number of subjects to be analyzed decreased by 4 men and 3 women for (a), for 4 men and 7 women (b) and 5 men and 8 women for (c) due to missing values for physical activity

**Table 3:** Odds ratios (95% CIs) for protein (a) or potassium (b) intake and risk of microalbuminuria

(a)	Quartile of protein intake				*P for trend*
Men	Q1 (< 60.3 g/day)	Q2 (60.3 to 69.6 g/day)	Q3 (69.7 to 78.5 g/day)	Q4 (≥78.6 g/day)	
Cases, n	146	156	161	167	
Controls, n	418	410	403	398	
Unadjusted	1.00 (reference)	1.09 (0.84 to 1.42)	1.14 (0.88 to 1.49)	1.20 (0.93 to 1.56)	0.1554
Model 1	1.00 (reference)	0.93 (0.71 to 1.23)	0.90 (0.68 to 1.18)	0.83 (0.63 to 1.09)	0.1738
Model 2	1.00 (reference)	1.09 (0.82 to 1.45)	1.09 (0.81 to 1.47)	1.01 (0.74 to 1.38)	0.9946
Model 3	1.00 (reference)	1.11 (0.82 to 1.50)	1.07 (0.78 to 1.47)	0.96 (0.69 to 1.35)	0.7172
Women	Q1 (< 65.0 g/day)	Q2 (65.0 to 71.8 g/day)	Q3 (71.9 to 79.9 g/day)	Q4 (≥80.0 g/day)	
Cases, n	154	158	186	207	
Controls, n	341	338	310	289	
Unadjusted	1.00 (reference)	1.04 (0.79 to 1.35)	1.33 (1.02 to 1.73)	1.59 (1.22 to 2.06)	< 0.0001
Model 1	1.00 (reference)	0.95 (0.71 to 1.28)	1.09 (0.82 to 1.45)	1.17 (0.88 to 1.55)	0.1824
Model 2	1.00 (reference)	0.995 (0.74 to 1.34)	1.09 (0.82 to 1.46)	1.21 (0.91 to 1.62)	0.1476
Model 3	1.00 (reference)	0.97 (0.71 to 1.34)	1.11 (0.81 to 1.51)	1.19 (0.87 to 1.63)	0.1830
(b)	Quartile of potassium intake				*P for trend*
Men	Q1 (< 2246.2 mEq/day)	Q2 (2246.2 to 2796.1 mEq/day)	Q3 (2796.2 to 3451.1 mEq/day)	Q4 (≥3451.2 mEq/day)	
Cases, n	168	157	146	159	
Controls, n	397	408	418	406	
Unadjusted	1.00 (reference)	0.91 (0.70 to 1.18)	0.83 (0.64 to 1.07)	0.93 (0.72 to 1.20)	0.4288
Model 1	1.00 (reference)	0.78 (0.59 to 1.02)	0.64 (0.49 to 0.84)	0.63 (0.48 to 0.83)	0.0005
Model 2	1.00 (reference)	0.84 (0.64 to 1.11)	0.70 (0.52 to 0.93)	0.70 (0.52 to 0.94)	0.0101
Model 3	1.00 (reference)	0.83 (0.62 to 1.12)	0.64 (0.47 to 0.88)	0.63 (0.44 to 0.89)	0.0035
Women	Q1 (< 2860.8 mEq/day)	Q2 (2860.8 to 3417.8 mEq/day)	Q3 (3417.9 to 4056.2 mEq/day)	Q4 (≥4056.3 mEq/day)	
Cases, n	191	179	172	163	
Controls, n	305	316	325	332	
Unadjusted	1.00 (reference)	0.91 (0.70 to 1.17)	0.85 (0.65 to 1.09)	0.78 (0.60 to 1.02)	0.0573
Model 1	1.00 (reference)	0.78 (0.59 to 1.04)	0.71 (0.53 to 0.94)	0.64 (0.48 to 0.85)	0.0021
Model 2	1.00 (reference)	0.80 (0.60 to 1.06)	0.71 (0.53 to 0.95)	0.67 (0.50 to 0.89)	0.0050
Model 3	1.00 (reference)	0.83 (0.61 to 1.13)	0.74 (0.54 to 1.02)	0.65 (0.46 to 0.92)	0.0116

Protein and potassium intake is calculated from a food frequency questionnaire and energy adjusted by the residual method

‘Controls’ means normoalbuminuric cases (ACR < 1.13 mg/mmol)

Model 1 was adjusted for age (years, continuous); Model 2 was adjusted as for model 1 plus body mass index (kg/m^2^, continuous), physical activity (metabolic equivalent task hour, continuous), smoking status (non-smoker, former smoker, or current smoker), alcohol consumption (< 150 g, 150–299 g, 300–449 g, > 450 g ethanol/week), energy intake (kcal/day, quartiles); and Model 3 was adjusted as for model 2 plus eGFR (ml/min/1.73 m^2^, continuous), dietary salt intake (g/day, continuous), pulse pressure (mmHg, continuous), diabetes (yes or no), and hypertention (yes or no)

In model 2 and model 3, the number of subjects to be analyzed decreased by 4 men and 3 women due to missing values for physical activity

## Results

Subjects were 991 men (30.5%) and 1451 women (42.2%) with high normoalbuminuria and 630 men (19.4%) and 705 women (20.5%) with microalbuminuria. Mean ± SD age was 69.2 ± 10.3 years in men and 68.3 ± 9.6 years in women; median (IQR) NEAP was 43.4 (34.2–53.4) mEq/day in men and 35.0 (27.7–43.6) mEq/day in women; median ACR (IQR) was 1.11 (0.57–2.49) mg/mmol in men and 1.47 (0.82–2.83) mg/mmol in women; and median eGFR (IQR) was 73.7 (64.1–84.3) ml/min per 1.73 m^2^ in men and 73.7 (64.7–83.7) ml/min per 1.73 m^2^ in women, respectively. Compared with women, men were older, were more frequently smokers, drinkers, hypertensive, and diabetic, had higher body mass index, total physical activity, systolic blood pressure, and diastolic blood pressure (*P* <  0.0001, respectively), and had lower pulse pressure (*P* = 0.0015). For dietary content, meat and egg intake did not differ between men and women.

Characteristics of the subjects including dietary content according to the quartile of NEAP are presented in Table 1. Higher NEAP was associated with higher ACR in both men and women, younger age, current smoker, greater alcohol consumption, and higher diastolic blood pressure in men, and with less total physical activity and hypertension in women. Regarding energy-adjusted nutrient intake, higher NEAP was associated with higher protein, in particular higher animal protein, and less plant protein, carbohydrate, potassium, calcium, magnesium, phosphorus, and salt intake in both men and women. Of the major food groups, fish, meat, grain, and egg intake was positively associated with higher NEAP, and vegetable and fruit intake was negatively associated with higher NEAP in both men and women.

The association between NEAP quartile and three grades of albuminuria, microalbuminuria, high normoalbuminuria, and high normoalbuminuria or microalbuminuria, was analyzed (Table 2). First, regarding for the presence of microalbuminuria versus controls (ACR < 1.13 mmol/mg) (*n* = 4242), higher NEAP quartile was associated with higher odds ratio in men (*P* for trend = 0.0130) and women (*P* for trend = 0.0014) in the fully adjusted model (as Model 3 in Table 2-a). Second, comparable multivariate logistic regression analysis was carried out in subjects without microalbuminuria (*n* = 5349). Regarding the presence of high normoalbuminuria, higher NEAP quartile was associated with higher odds ratio in women (*P* for trend = 0.0163), but not men (*P* for trend = 0.2391) (Model 3 in Table 2-b). Third, regarding the presence of high normoalbuminuria or microalbuminuria, higher NEAP quartile was associated with higher odds ratio in men (*P* for trend = 0.0407) and women (*P* for trend = 0.0028), (as Model 3 in Table 2-c). Similar analysis was performed using PRAL as an alternative. The fully adjusted odds ratio (Model 3) for the presence of microalbuminuria, high normoalbuminuria, and high normoalbuminuria or microalbuminuria comparing the lowest to the highest PRAL quartile was 1.39 (95% CI: 1.03–1.90, *P* for trend = 0.0338), 1.16 (95% CI: 0.91–1.49, *P* for trend = 0.2375), and 1.24 (95% CI: 0.99–1.55, *P* for trend = 0.055) in men, and was 1.48 (95% CI: 1.06–2.07, *P* for trend = 0.003), 1.34 (95% CI: 1.05–1.72, *P* for trend = 0.0116), and 1.37 (95% CI: 1.09–1.72, *P* for trend = 0.0049) in women, respectively.

In terms of analysis of the association between nutrient components associated with NEAP and albuminuria, similar multivariate logistic analyses were implemented to calculate the adjusted odds ratios for microalbuminuria according to protein or potassium quartile (Table 3). Protein intake was not associated with microalbuminuria in either men or women, and neither animal protein intake nor plant protein intake was associated with microalbuminuria (data not shown). In contrast, a higher quartile of potassium intake was associated with a lower odds ratio for microalbuminuria. The adjusted odds ratio for the presence of high normoalbuminuria or microalbuminuria when comparing the lowest and highest potassium quartiles was 0.73 (95% CI: 0.57–0.94, *P* for trend = 0.0094) in men and 0.75 (95% CI: 0.59–0.95, *P* for trend = 0.0304) in women; protein intake, including animal and plant protein, had no significantly lower or higher adjusted odds ratio for this grade of albuminuria.

## Discussion

This study showed the association between dietary acid load and albuminuria as previously reported; Banerjee et al. reported the association between estimated net acid excretion, using net acid excretion (calculated using PRAL and organic acids) derived from estimated nutrient intake data based on dietary recall questionnaire, and albuminuria based on cross-sectional data from the National Health and Nutrition Examination Survey (NHANES) 1999–2004 in adults in the US [4]. They also reported the association between high dietary acid load using net acid excretion and progression to end-stage renal disease (ESRD) among adult CKD patients with albuminuria [25]. From the Jackson Heart Study, conducted in a community-based African-American population in the US, higher net acid excretion derived from FFQ information was independently associated with the presence of microalbuminuria [5]. Regarding the association between NEAP and albuminuria, the Atherosclerosis Risk in Communities study, a community-based observational study in middle-aged adults in the US, reported that higher NEAP was associated with incident CKD [26]. Also, other studies from East Asia found that higher NEAP was associated with CKD progression in elderly populations [6, 14].

However, it should be noted that these previous studies defined albuminuria as ACR ≥3.89 mg/mmol, a cutoff value that is higher than that in our study (1.13 mg/mmol). The findings of the present study reveal the association between increasing NEAP and high normoalbuminuria in women. A similar but weaker association was seen in men. Also, compared with women, men had lower albuminuria and tended to have hypertension and diabetes. This suggests a sex difference in the association between increasing NEAP and high normoalbuminuria. A novel finding of our study was the association between estimated dietary acid load and high normoalbuminuria, and to our knowledge, this study is the first report of such an association. The clinical relevance of high normoalbuminuria has been suggested because it could lead to adverse outcomes such as cardiovascular disease, ESRD, and all-cause death in the general population [8, 9, 27]. Furthermore, for primary prevention of albuminuria, it is important to identify lifestyle-factors associated with high normoalbuminuria or microalbuminuria.

We investigated the nutrients related to NEAP and found that potassium was an important dietary component for the association between NEAP and albuminuria, but protein was not. Some other studies reported an association of potassium intake with CKD. Ko et al. reported an association between dietary acid load and CKD in community-dwelling elderly Koreans (*n* = 1369, aged ≥65 years) and that potassium intake was associated with CKD, but protein intake was not [6]. Additionally, another Japanese study (*n* = 217) reported that potassium excretion, but not protein, in 24-h urine samples was a significant component of NEAP [14]. Although the results of these studies were similar to the present study finding in terms of nutrients and NEAP, the present study is complemented with a larger-scale compared with these previous studies. Also, these findings from East Asian countries may suggest that the role of potassium in the association of NEAP with CKD including albuminuria depends on food preference. Because fruits and vegetables are major sources of potassium, some studies have reported their significance [4, 28]. An interventional study confirmed that kidney injury decreased following reduced acid load in humans, and that acid load reduction by consumption of fruits and vegetables decreased kidney injury markers, including urine albumin concentration, in hypertensive CKD patients with low eGFR (eGFR 60–89 ml/min/1.73 m^2^ or eGFR 15–29 ml/min/1.73 m^2^) [28]. The NHANES III study also found that high consumption of fruits and vegetables was inversely associated with albuminuria [4].

In terms of acid-base balance, protein contributes to acid production due to the content of sulfur-containing amino acids. According to Remer et al. evaluating the acid-forming potential of more than 100 frequently consumed foods and beverages by assessing PRAL revealed that animal protein was a source of higher acid production than plant protein [21]. In the present study, total protein intake and animal protein intake was positively associated with increasing NEAP and plant protein intake was inversely associated with increasing NEAP (Table 1); however, the association between animal or plant protein intake and albuminuria was not significant. Although a previous study from the US reported the average American dietary protein resource as being predominantly from animal sources (69%) [29], the present study showed that the proportion of animal protein in total protein intake was an average of 46.2% in men and 49.4% in women. Thus, intake of animal protein might have less impact on the association between NEAP and albuminuria than reported in Western countries.

To our knowledge, there is limited nutritional epidemiologic data aimed at reducing albuminuria. Our findings may indicate that protein restriction has a less important role but potassium has a more important role in individuals, especially East Asians, with high normoalbuminuria or microalbuminuria. However, an important note is that our study did not provide evidence on the safety limit for potassium or protein intake.

Our study has some limitations. First, ACR was measured only once and this might cause misclassification of albuminuria and misunderstanding as to whether there would be chronicity. Furthermore, there was no information as to the type of antihypertensive medication such as renin-angiotensin system blockers. Thus, the effect of drugs which would possibly affect the degree of albuminuria could not be fully eliminated. Second, measurements of plasma pH or HCO_3_ which are indices of the degree of acidemia were not available. Third, dietary acid load was estimated using NEAP from self-reported FFQ information only. Dietary contents could be assessed by only a semi-quantitative evaluation. In addition, our FFQ was validated by comparing a 12-day weighed food record in men and women aged 40–74 years (*n* = 240) [20], but the subjects in the present study ranged in age from 40 years up to 97 years old. This difference in validation might cause misclassification of exposures and could weaken the strength of association. Fourth, because of the cross-sectional observational study design, a causal relationship between dietary acid load and albuminuria cannot be ascertained. Fifth, the subjects were from the Uonuma area of Japan, and so these findings may not be generalizable to other East Asian populations or other ethnicities worldwide.

## Conclusion

In conclusion, our study has shown that higher dietary acid load was associated with the presence of not only microalbuminuria but also high normoalbuminuria in an adult Japanese population. Regarding the nutrients associated with dietary acid load, potassium intake was negatively associated with the early stages of albuminuria. Longitudinal studies are needed to confirm whether dietary acid load influences the development and progression of albuminuria.

## Abbreviations

95% CI: 95% confidence interval
ACR: Urine albumin-to-creatinine ratio
BMI: Body mass index
CKD: Chronic kidney disease
eGFR: Estimated glomerular filtration rate
ESRD: End-stage renal disease
FFQ: Food frequency questionnaire
HbA1c: Glycated hemoglobin
IQR: Interquartile range
NEAP: Net endogenous acid production
NHANE: National Health and Nutrition Examination Survey
PRAL: Potential renal acid load
